# Caffeine Combined with Excitatory Neuromodulation Based on Transcranial Direct Current Stimulation (tDCS) Enhances Performance in a Time-Trial CrossFit^®^ Workout: A Randomized, Placebo-Controlled, Double-Blind Study

**DOI:** 10.3390/nu17071261

**Published:** 2025-04-03

**Authors:** Alberto Souza Sá Filho, Thiago Albernaz-Silva, Pedro Augusto Inacio, Vicente Aprigliano, Iransé Oliveira-Silva, Gaspar R. Chiappa, Rodolfo P. Vieira, Antônio Sérgio Nakao de Aguiar, Raphael Martins Cunha, James Oluwagbamigbe Fajemiroye, Marcelo Magalhães Sales

**Affiliations:** 1 Department of Human Movement and Rehabilitation (PPGMHR) and Pharmaceutical Sciences, Pharmacology and Therapeutics (PPGCFFT), Graduate Program at the Evangelical University of Goiás (UniEVANGÉLICA), Anápolis 75083-515, GO, Brazil; alberto.filho@unievangelica.edu.br (A.S.S.F.); thiagoalbernaz@yahoo.com.br (T.A.-S.); pedroqinacio@gmail.com (P.A.I.); iranse.silva@unievangelica.edu.br (I.O.-S.); gaspar.chiappa@gmail.com (G.R.C.); rodrelena@yahoo.com.br (R.P.V.); antonio.aguiar@docente.unievangelica.edu.br (A.S.N.d.A.); prof.raphaelcunha@gmail.com (R.M.C.); jamesfajemiroye@ufg.br (J.O.F.); 2 Escuela de Ingeniería de Construcción y Transporte, Pontificia Universidad Católica de Valparaíso, Avda Brasil 2147, Valparaíso 2362804, Chile; 3 Faculty of Health Sciences, Universidad Autónoma de Chile, Providencia, Santiago 7500912, Chile; 4 Institute of Biological Sciences, Federal University of Goiás, Goiânia 74690-900, GO, Brazil; 5 Graduate Program in Environmental and Society, Academic Institute of Health and Biological Sciences, State University of Goiás, Southwest Campus, Quirinópolis 75862-196, GO, Brazil; marcelomagalhaessales@gmail.com

**Keywords:** tDCS, performance, HIFT, HIIT, high-intensity functional training, CrossFit^®^

## Abstract

**Background:** Caffeine (CAF) and transcranial direct current stimulation (tDCS) are ergogenic strategies with potential benefits for performance, yet their combined effects remain underexplored, particularly in high-intensity functional training contexts such as CrossFit^®^. **Objective:** This randomized, double-blind, placebo-controlled crossover study aimed to investigate the impact of tDCS, with and without CAF, on performance time in the Clean & Jerk (C&J) during the benchmark WOD GRACE among competitive CrossFit^®^ athletes. Secondarily, we aimed to compare the RPE across the different experimental conditions, as well as to establish the relationship between personal record (PR) values adjusted for body mass and the execution time of the WOD GRACE, considering different athletes’ classification levels (RX Elite and RX Intermediate). **Methods:** Twenty participants completed four experimental conditions: CAF ingestion (400 mg) combined with anodal tDCS (CAF + a-tDCS), CAF with Sham tDCS (CAF + Sham-tDCS), placebo (PLA) with a-tDCS (PLA + a-tDCS), and PLA with Sham tDCS (PLA + Sham-tDCS). **Results:** The results indicated that the combination of CAF + a-tDCS significantly improved performance, reducing execution time (205.5 ± 58.0 s) compared to CAF + Sham-tDCS (218.3 ± 61.2 s; *p* = 0.034), PLA + a-tDCS (231.7 ± 64.1 s; *p* = 0.012), and PLA + Sham-tDCS (240.9 ± 66.4 s; *p* = 0.002). However, no significant differences were observed between CAF + Sham-tDCS and PLA + a-tDCS (*p* = 0.690), CAF + Sham-tDCS and PLA + Sham-tDCS (*p* = 0.352), or PLA + a-tDCS and PLA + Sham-tDCS (*p* = 0.595). The responder analysis revealed that 45% of participants improved performance with isolated tDCS, while 60% responded positively to CAF. No significant differences were found in RPE scores among conditions (*p* = 0.145). Additionally, no correlations were identified between PR values adjusted for body mass and execution time in both RX Elite (r = 0.265; *p* = 0.526) and RX Intermediate (r = 0.049; *p* = 0.901) groups, nor between training experience and performance across interventions. **Conclusions:** These findings suggest that tDCS, when combined with CAF, may serve as an effective ergogenic aid for improving performance in high-intensity functional training, whereas its isolated use does not yield meaningful benefits.

## 1. Introduction

The use of ergogenic aids to enhance athletic performance has been widely explored in sports science, particularly in high-intensity modalities such as CrossFit^®^. Among the various strategies available, caffeine (CAF) supplementation and transcranial direct current stimulation (tDCS) have gained significant attention due to their potential impact on physical and cognitive performance [1,2]. CAF is a well-established ergogenic aid, known for its ability to reduce the rating of perceived exertion (RPE), increase muscle strength, and delay fatigue by acting on adenosine receptors in the central nervous system [2,3].

In parallel, tDCS has emerged as a non-invasive brain stimulation technique capable of enhancing cortical excitability, thereby improving motor function and endurance performance [1,4]. On the other hand, tDCS, has been widely investigated for its effects on performance [5,6]. Studies have shown that anodal tDCS (a-tDCS) applied over the primary motor cortex (M1) can enhance force production, delay neuromuscular fatigue, and improve motor coordination, leading to increased endurance, strength performance [4,7,8], and reduced RPE [6,9]. A systematic review by Machado et al. [10] indicated that a-tDCS significantly improves exercise performance in tasks requiring maximal voluntary contractions, although its effects on muscle endurance remain less conclusive.

CrossFit™ workouts incorporate a combination of aerobic and anaerobic demands, requiring both neuromuscular efficiency and metabolic conditioning [11]. Given the physiological complexity of CrossFit™, interventions that optimize neuromuscular function and delay fatigue could offer a competitive advantage, especially in short-duration strength workouts such as the benchmark GRACE [12]. The study by Meier et al. [13] analyzed CrossFit™ performance benchmarks and found that GRACE performance (30 reps of Clean and Jerk—C&J) varies significantly based on an athlete’s strength and Olympic weightlifting proficiency, whereas male athletes completed GRACE with an average time of 233.3 ± 101.2 s, while female athletes had an average time of 250.6 ± 171.2 s. Previous research on CrossFit™ performance has focused on various supplementation strategies, including CAF and sodium bicarbonate, yet findings remain inconsistent [14,15]. Additionally, only one study has specifically looked at the GRACE workout [16].

Despite the well-documented independent effects of CAF and tDCS on performance [10,17,18], few studies have examined their combined effects in high-intensity functional training contexts [6,19]. Theoretical models suggest that CAF and tDCS may exert complementary effects, as CAF enhances neuromuscular activation and cognitive alertness, while tDCS facilitates motor cortex excitability and muscular endurance [1,6,7,10]. Some studies have reported improved performance and reduced RPE following CAF ingestion, while others have failed to demonstrate significant effects on muscular power or endurance [19]. Additionally, tDCS application in elite athletes has yielded mixed results, with some studies reporting significant performance enhancements, whereas others have shown minimal impact [7,20]. This discrepancy underscores the need for further investigation into the combined effects of CAF and tDCS in real-world athletic settings [20].

Given the central role of neuromuscular efficiency in these activities, the integration of tDCS and CAF may offer a novel strategy for performance enhancement [19,20]; there is a significant gap in the literature that remains largely unexplored. The primary objective of the present study was to investigate the effects of tDCS, with and without CAF supplementation, on execution time in the C&J exercise (benchmark WOD GRACE) among competitive CrossFit™ athletes. Additionally, we identified responders and non-responders to tDCS and CAF, evaluating their influence on physical performance.

Secondarily, we aimed to compare the RPE across the different experimental conditions, as well as to establish the relationship between personal record (PR) values adjusted for body mass (BM) and the execution time of the WOD GRACE, considering different athletes’ classification levels (RX Elite and RX Intermediate).

As the primary hypothesis, we hypothesized that tDCS alone would produce significant effects compared to the other conditions. Furthermore, CAF would enhance the effects of tDCS, substantially improving performance. Secondly, we believe that tDCS, or tDCS in combination with CAF, will produce lower RPE. Additionally, more than half of the participants will respond to both tDCS and CAF interventions. Finally, RX Elite participants, who exhibit a higher level of strength relative to BM, were expected to achieve superior performance in the WOD GRACE, with their results being associated with PR values.

## 2. Methods

### 2.1. Experimental Approach

This is a randomized, double-blind, placebo-controlled crossover trial with a repeated-measures design. The present study was conducted in accordance with the principles outlined by the International Committee of Medical Journal Editors (ICMJE) [21] and was designed based on the STROBE Statement guidelines for observational studies, specifically for analytical cross-sectional studies [22], and was conducted over a total of six visits. The study adhered to Resolution 466/2012 of the National Health Council and was approved by the Research Ethics Committee (CAAE 25686219.9.0000.5512; protocol 3.790.808), as well as the Declaration of Helsinki regarding ethical principles for clinical research involving human subjects.

After selection, acceptance, and full comprehension of the inherent risks of high-intensity exercise, all participants signed the informed consent form and voluntarily agreed to participate in the study.

### 2.2. Participants

A total of 30 male participants responded to the public calls for voluntary participation in this study. All participants were recruited through a public call process conducted in two stages across three different CrossFit™ boxes located in the Midwest region of Brazil (capital and surrounding areas) between June 2023 and February 2024. All participants were physically active and classified as advanced or highly advanced in multimodal exercise [23], with over five years of training experience. The classification of the sample participants can be observed in the Section 3.1 within the Section 3.

Participants were included based on the following criteria: competitively trained in CrossFit™ for more than one year on a regular basis and non-smokers. Exclusion criteria included individuals who used psychoactive or prior ergogenic substances or had musculoskeletal injuries that could limit performance assessments.

The participants completed the risk stratification questionnaire for coronary artery disease (CAD), as proposed by the American College of Sports Medicine [24]. All individuals participating in the study were verbally informed and signed a written informed consent form.

### 2.3. Study Design

This study was conducted over a total of six visits. In the first session, after signing the informed consent form, all participants underwent a bioelectrical impedance analysis to characterize the sample and completed the CAD risk stratification questionnaire. At this time, the athletes’ training level was also determined via an interview, based on the criteria of Santos-Junior et al. [23]. Subsequently, participants performed the determination of PR values. Although participants routinely performed the WOD GRACE during their training sessions, a familiarization session was required to establish their pacing strategy.

From the second to the sixth visit, participants underwent experimental procedures, which consisted of the following conditions: (a) ingestion of 400 mg of CAF (CAF) + a-tDCS (a-tDCS); (b) ingestion of 400 mg of placebo (PLA) + a-tDCS; (c) ingestion of 400 mg of CAF + Sham-tDCS; (d) ingestion of 400 mg of PLA + Sham-tDCS.

**Figure 1:**
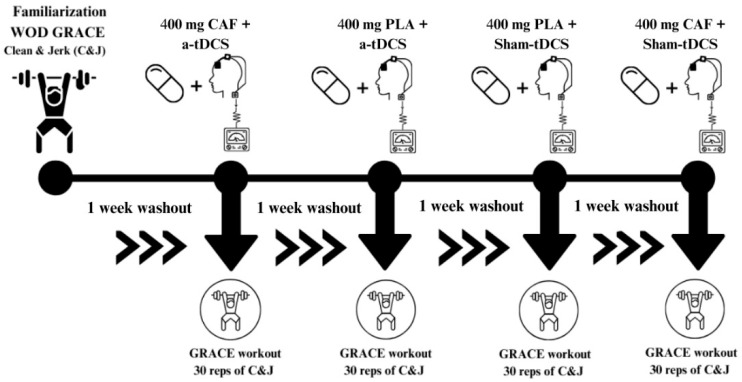
Design of the study. CAF = ingestion of 400 mg of caffeine; a-tDCS = anodal transcranial direct current stimulation; PLA = placebo substance (calcium carbonate); Sham = Sham-tDCS (placebo) condition; C&J = Clean and Jerk.

All conditions were randomly assigned through a lottery-based selection. Both CAF and PLA were administered in a double-blind manner, 20 min before the initiation of the tDCS procedure. The tDCS protocol lasted 20 min, totaling 40 min from ingestion to the start of the experimental task. The C&J task was performed identically in each session, requiring a total of 30 valid repetitions. The exercise time was recorded, along with the RPE. Figure 1 illustrates the experimental design of the study.

### 2.4. Outcome Variables

The primary outcome measure of the study is performance time in the WOD GRACE. This is expressed as total execution time in seconds, representing the time taken to complete 30 repetitions of C&J under different experimental conditions. Secondarily, C&J performances across visits were related to PR values. The RPE was also compared.

### 2.5. Procedures

#### 2.5.1. Bioimpedance

Total BM and body composition were assessed using a multi-frequency bioelectrical impedance scale, InBody^®^ (model 230, Biospace, Republic of Korea). The bioelectrical impedance analysis (BIA) was conducted following the standardized protocol described by Ellis et al. [25], which required participants to fast for at least four hours before the test, refrain from engaging in intense physical activity for at least 24 h, urinate at least 30 min before the assessment, abstain from alcohol consumption for 48 h, avoid the use of diuretic medications for seven days, and remain in a supine position for at least 5 to 10 min in total rest before the test. During the assessment, participants stood barefoot on the BIA scale and held the hand electrodes, allowing for a low-amperage electrical current to pass through the body. This method enabled the estimation of body fat percentage (Fat%), lean mass (kg), and total body water (L).

#### 2.5.2. Personal Record

To determine the PR values, two distinct strategies were employed: (a) PR values previously recorded by the coach and (b) PR values measured during the experimental session. The previously recorded PR values were extracted from daily tracking applications and considered only for the most experienced and highest-performing athletes (completion time < 3 min for GRACE), provided they had been recorded within the last month. For the remaining athletes, a preliminary session prior to the WOD GRACE experimental procedure was conducted to determine their PR values. For this standardized collection, following the mobility and progressive loading phase, all participants were given a total of 420 s to perform three attempts at achieving their maximum one-rep C&J.

#### 2.5.3. Participant Categorization—RX (as Prescribed)

Participants were classified as RX if they completed their protocols using the officially prescribed loads according to CrossFit™ standards, as outlined in the experimental design. The sample was further stratified into two RX categories based on performance time: (a) RX Elite, for those who completed the protocol in less than 3 min, and (b) RX Intermediate, for those with a performance time exceeding 3 min.

#### 2.5.4. Transcranial Direct Current Stimulation (tDCS) Protocol

Participants remained comfortably seated in a chair within the CrossFit^®^ box training during the tDCS application. A constant electrical current of 2 mA was applied using a pair of saline-soaked electrodes (140 mmol NaCl dissolved in Milli-Q water), each measuring 35 cm^2^. The anodal and cathodal electrodes were connected to a continuous current stimulation device powered by three 9V batteries, which were calibrated using a digital multimeter (EZA EZ 984, Xi’an, China). For the a-tDCS, the anodal electrode was positioned over the left dorsolateral prefrontal cortex (DLPFC), corresponding to the F3 electrode site as per the 10–20 International EEG System, and the cathode was positioned in the orbitofrontal region (FP2).

In the Sham-tDCS (placebo) condition, the electrodes were placed in the same positions as in the a-tDCS stimulation protocol. However, the stimulation device was programmed to gradually decrease the current and switch off after 60 s, a method previously reported as ineffective stimulation. This placebo procedure ensures that participants may still perceive initial sensations such as tingling or mild itching due to the brief electrical stimulation, yet do not receive an actual sustained current. This approach effectively maintains blinding, preventing participants from distinguishing between real and placebo stimulation conditions, thereby ensuring an appropriate control effect.

**Figure 2:**
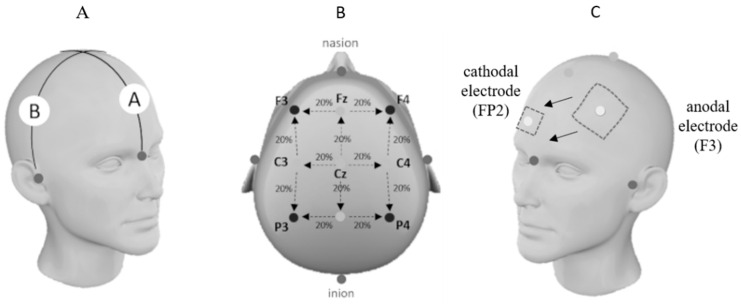
The tDCS electrodes assembly pattern. In the left image (**A**), the initial process for segmentation according to the 10/20 EEG reference system is illustrated; in the central image (**B**), the procedure to identify the reference area corresponding to the left dorsolateral prefrontal cortex (left DLPFC) is presented; in the right image (**C**), the placement of both electrodes over the targeted stimulation sites is shown.

The electrode assembly was the same in all conditions. The a-tDCS and Sham-tDCS conditions were applied for a time of 20 min. Figure 2 illustrates the tDCS electrodes assembly pattern.

#### 2.5.5. Caffeine (CAF) Supplementation

A dose of 400 mg of anhydrous CAF or 400 mg of a placebo substance (calcium carbonate) was randomly administered orally in the form of white gelatin capsules. The principal investigator provided the capsule 20 min before the tDCS procedure, which lasted 20 min, totaling 40 min before the exercise. The selection process (CAF or PLA) was conducted in a double-blind manner, ensuring that neither the participants nor the principal investigator were aware of the assigned condition.

Participants were instructed to refrain from consuming any CAF-containing foods or coffee on the day of data collection. All ergogenic resources used in this study are publicly available and do not require medical prescriptions. CAF administration followed sports supplementation guidelines in accordance with the position statement of the International Society of Sports Nutrition [26].

#### 2.5.6. Experimental Protocol for C&J

The protocol began with a sequence of joint mobility exercises targeting the hip, ankle, thoracic spine, shoulder, and wrist, followed by a few repetitions of the C&J movement with progressive loading (5 to 10 repetitions) to prepare participants for the main task. The entire preparation phase lasted ten minutes and was performed at a moderate perceived intensity, ensuring that the warm-up did not interfere with the outcome variables.

Following the warm-up, participants performed the WOD GRACE, executing a total of 30 repetitions of the C&J in the shortest possible time, with a fixed workload of 135 lbs for men. The C&J movement began with the barbell resting on the ground. The athlete lifted the barbell in a continuous motion to the front rack position, receiving it either in a full squat or in a power position with up to 100° of knee flexion. In the second phase of the movement, after achieving full knee and hip extension, the athlete moved the barbell from the front rack position to a fully extended overhead position using one of the accepted jerk techniques, such as the push jerk, power jerk, or squat jerk.

A repetition was only considered valid when the barbell was securely locked out overhead, with the elbows fully extended and the feet realigned under the hips. Any deviation from the established movement standards or failure to fully extend the arms was classified as a No Rep. However, a loss of control after completing the repetition was still considered a valid attempt as long as the movement was fully executed.

In order to standardize the C&J movement, it was established that participants could not perform the base change movement after receiving the Olympic bar (Split-Jerk). The trial pacing strategy was individually determined by each participant, allowing them to release the bar at their discretion whenever deemed most appropriate.

**Figure 3:**
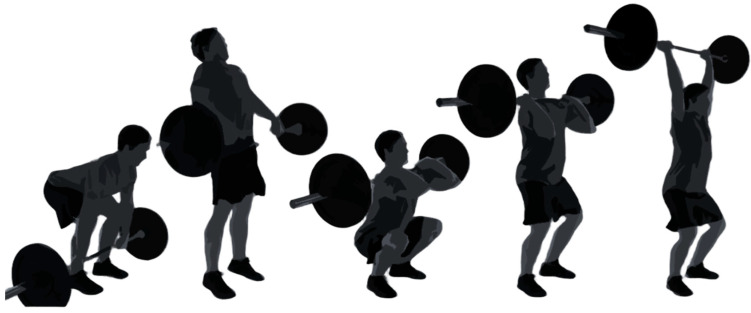
Clean & Jerk (C&J) lifting movement pattern.

To maintain sample homogeneity, the maximum execution time was set at 360 s. The participants had access to the timer at all times. The total execution time of the protocol was recorded in seconds. The RPE was recorded 10 min after the completion of the session. Figure 3 illustrates the complete Clean and Jerk (C&J) movement.

#### 2.5.7. Rating of Perceived Exertion (RPE) Scale

The adapted linear RPE scale (CR 0–10), as developed and described by Borg and adapted by Foster et al. [27], was used to assess perceived exertion. In this scale, “0” corresponds to a perception of “extremely light” effort, while “10” represents “total fatigue”. As this is a short- to medium-duration task, the session RPE was assessed 10 min after the completion of the WOD GRACE.

### 2.6. Allometry

The BM variable was adjusted using the standard exponent, scaling relative strength to 1.0 (kg/BM). The association analyses examining the influence of relative strength on performance were conducted for the RX Elite and RX Intermediate groups.

### 2.7. Study Size

The sample size was determined based on the following parameters: F-test—ANOVA repeated measures, within-factor; Analysis Criterion: Compute required alpha; Effect size: 0.4; Power: 0.95; Total sample size: 20 participants; Number of groups: 1; Number of measurements: 4. The following output parameters were obtained: Critical F: 3.762; Alpha error probability: 0.001.

### 2.8. Randomization Process

Session randomization was performed in an attempt to minimize the effect of learning on outcome variables. To this end, a computer-generated random sequence was used to assign the different exercise sessions, i.e., (a) ingestion of 400 mg of CAF + a-tDCS; (b) ingestion of 400 mg of PLA + a-tDCS; (c) ingestion of 400 mg of CAF + Sham-tDCS; and (d) ingestion of 400 mg of PLA + Sham-tDCS at each laboratory visit. Session selection at each laboratory visit was performed by an independent researcher who was not involved in data collection or analysis, ensuring a double-blind design. Participants and evaluators remained blinded to exercise session assignments until completion of statistical analyses.

### 2.9. Risk of Bias, Blinding, and Data Processing

To minimize potential analysis bias, data collection was conducted by two independent researchers affiliated with the project and research group (A.R. and T.A.) and subsequently analyzed by a third researcher (research group leader, A.S.). Given the double-blind study design, one evaluator was responsible for blinding both the participants and the principal investigator at the time they received the CAF capsule or placebo. To ensure that both the participants and the principal investigator remained blinded to the type of tDCS intervention administered, the stimulation device was concealed throughout the 20 min intervention period. Finally, the third researcher received and analyzed the data in a blinded manner, with interventions coded numerically. The group allocations were only revealed after the statistical output was generated.

### 2.10. Statistical Analysis

Data normality and homogeneity were assessed using the Shapiro-Wilk test and Levene’s test, respectively. Variables following a Gaussian normal distribution were presented as mean ± standard deviation (SD) and 95% confidence interval (CI^95%^). Non-normally distributed data were analyzed using non-parametric methods and reported as median and CI^95%^. Additionally, the analysis of dependent variables was performed using the Friedman test for related non-parametric samples. Responders and non-responders to the tDCS and CAF procedures were identified through visual graphical inspection. Pearson’s correlation was used to analyze the association between PR values relative to BM and the best performance. Finally, the effect size (ES) was calculated based on the equation $r=Z÷\;\sqrt{N}$, for non-parametric comparisons. All statistical analyses were conducted using the Statistical Package for the Social Sciences (SPSS, Version 23.0, for Windows^®^, Chicago, IL, USA). Sample size determination was performed using G*Power (Version 3.1.9.7), and graphical representations were generated using GraphPad Prism (Version 8.0). A significance level of 5% (*p* = 0.05) was adopted.

## 3. Results

### 3.1. General Information

After analyzing the statistical assumptions, the normality of the data was verified using the Shapiro-Wilk test for the demographic variables (experience time—*p* = 0.183; age—*p* = 0.940), anthropometric and body composition variables (BM—*p* = 0.095; height—*p* = 0.329; lean mass—*p* = 0.167; intracellular water—*p* = 0.123). However, the body mass index (BMI) (*p* = 0.038) and Fat% (*p* = 0.018) did not exhibit a normal distribution, and were therefore analyzed using non-parametric statistical methods.

**Figure 4:**
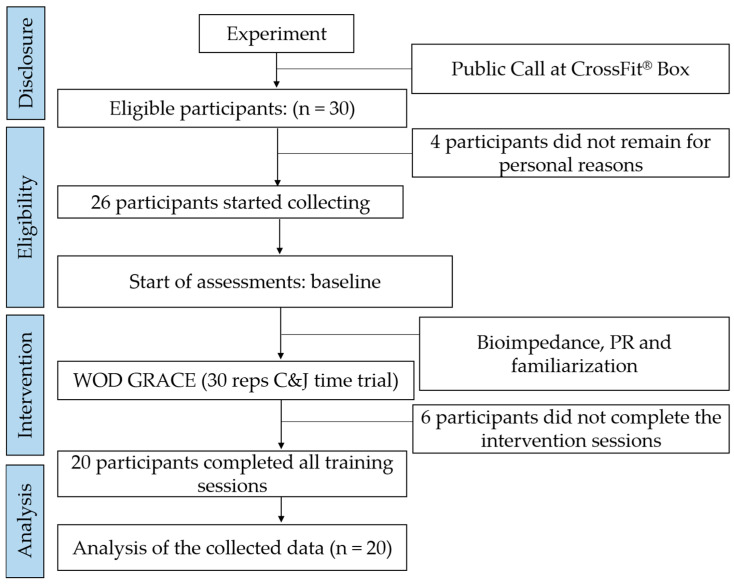
Participant inclusion and exclusion process. C&J = Clean & Jerk.

For the main intervention data, a normal distribution was observed for the PLA + Sham-tDCS (*p* = 0.176) and CAF + a-tDCS (*p* = 0.408) groups. However, normality was not confirmed for the PLA + a-tDCS (*p* = 0.023) and CAF + Sham-tDCS (*p* = 0.041) groups. Therefore, the main dependent variables were analyzed using non-parametric statistical methods. The distribution analysis of RPE scores was tested for each type of intervention, with no normality being confirmed in any of the cases (*p* < 0.001). Figure 4 illustrates the participant inclusion and exclusion process, while Table 1 presents the athletes’ characteristics.

### 3.2. Physical Performance

The classification of the athletes’ training level followed the recommendations proposed by Santos-Junior et al. [23]. The athletes achieved scores above 15 points based on the following criteria: (a) current uninterrupted training duration, (b) time of detraining, (c) previous training experience, (d) exercise technique, and (e) strength level. Therefore, a minimum average score of 3.0 points was required across the evaluated criteria, classifying them as “Advanced” to “Highly Advanced”.

The PR values demonstrated an average of 100.2 ± 11.1 kg, with the maximum external load lifted in WOD GRACE corresponding to 60.8% of PR. The best performance time in the WOD GRACE among the study participants was 205.5 ± 58 s.

### 3.3. Participant Categorization and Allometry

Among the participants involved in the sample, eight athletes were qualified as RX Elite (155.0 ± 21.1 s), while the others were qualified as RX Intermediate (250.6 ± 38.6 s). When adjusted for BM, athletes in the RX Elite category had a slightly higher level of strength (1.4 ± 0.2 kg/kg), while athletes classified as RX Intermediate had a lower level of relative strength (1.3 ± 0.1 kg/kg).

### 3.4. Caffeine (CAF) Administration

Each athlete was administered a total of 400 mg of CAF, providing an average dose of 5.2 ± 0.6 mg/kg (95% CI: 4.9 to 5.7 mg/kg; minimum: 4.0 mg/kg; maximum: 6.5 mg/kg).

### 3.5. Primary Outcome

#### 3.5.1. Comparison Between the Different Interventions

**Figure 5:**
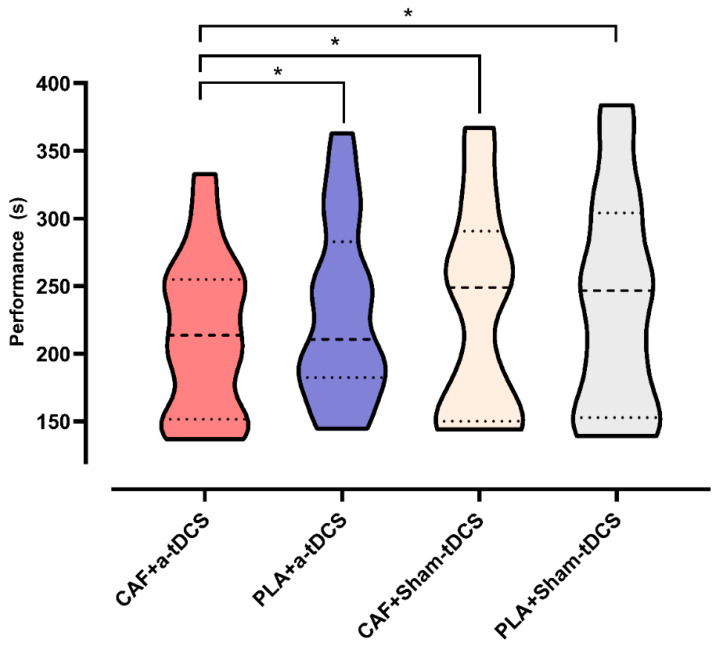
Differences among the experimental procedures. CAF + a-tDCS = ingestion of 400 mg of caffeine plus anodal transcranial direct current stimulation; PLA + a-tDCS = ingestion of 400 mg of placebo substance (calcium carbonate) plus anodal transcranial direct current stimulation; CAF + Sham-tDCS = ingestion of 400 mg of caffeine plus placebo condition for transcranial direct current stimulation; PLA + Sham-tDCS = ingestion of 400 mg of placebo substance (calcium carbonate) plus placebo condition for transcranial direct current stimulation. (*) Differences between the CAF + a-tDCS interventions and the other interventions.

**Figure 6:**
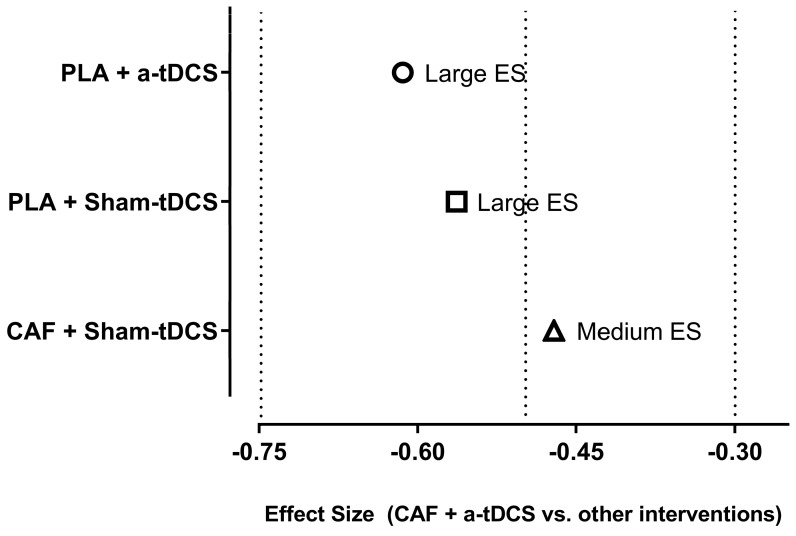
Magnitude of differences between CAF + a-tDCS and other interventions. Effect size (ES) for the condition combining caffeine supplementation and anodal transcranial direct current stimulation (CAF + a-tDCS) compared to the other interventions (PLA + a-tDCS, PLA + Sham-tDCS, and CAF + Sham-tDCS). Effect sizes were calculated based on Z-scores from the non-parametric analysis. Symbols represent the ES for each comparison: circle (PLA + a-tDCS), square (PLA + Sham-tDCS), and triangle (CAF + Sham-tDCS). Dashed vertical lines indicate the thresholds for ES classification, with values considered large (ES ≤ −0.60) or medium (−0.60 < ES ≤ −0.40). Negative values indicate greater effectiveness of the CAF + a-tDCS intervention.

The Friedman test for N related samples was conducted to compare the medians of the four experimental interventions, including CAF + a-tDCS, PLA + a-tDCS, CAF + Sham-tDCS, and PLA + Sham-tDCS, followed by a post hoc test to identify specific differences. The results indicated a significant difference among the experimental interventions (*p* = 0.013). Further post hoc analysis revealed that CAF + a-tDCS demonstrated significant differences when compared to CAF + Sham-tDCS (*p* = 0.034), PLA + a-tDCS (*p* = 0.012), and PLA + Sham-tDCS (*p* = 0.002). However, no significant differences were observed between CAF + Sham-tDCS and PLA + a-tDCS (*p* = 0.690), CAF + Sham-tDCS and PLA + Sham-tDCS (*p* = 0.352), or PLA + a-tDCS and PLA + Sham-tDCS (*p* = 0.595). Figure 5 illustrates the differences among the experimental procedures. The ES for the comparisons between interventions that supported the alternative hypothesis (*p* < 0.05) is presented in Figure 6.

#### 3.5.2. Responders and Non-Responders to the tDCS and CAF Interventions

**Figure 7:**
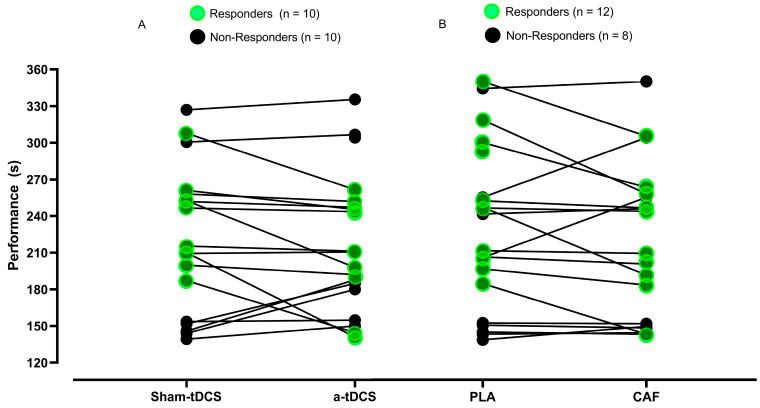
Responders and non-responders to the tDCS and CAF interventions; (**A**) represents responders to tDCS intervention; (**B**) represents responders to CAF intervention. CAF = ingestion of 400 mg of caffeine; a-tDCS = anodal transcranial direct current stimulation; PLA= placebo substance (calcium carbonate); Sham = Sham-tDCS (placebo) condition.

The analysis of responders and non-responders to the tDCS intervention was conducted considering the PLA + a-tDCS, meaning active tDCS without the influence of caffeine (CAF). Similarly, the analysis of responders to CAF was performed using the CAF + Sham-tDCS in the percentage-based evaluation. In both intervention methods, the PLA + Sham-tDCS condition was used as the reference point. Regarding tDCS, only 50% (*n* = 10) of the sample responded positively to the intervention, showing a reduction in total execution time. In contrast, for the CAF intervention, 60% (*n* = 12) of the participants exhibited a positive response following the ingestion of 400 mg of CAF. Figure 7 illustrates the responders and non-responders to the tDCS and CAF interventions. When the results were transmitted to the participants through an informal interview, but based on a semi-structured questionnaire (Supplementary Materials), the results indicated that among the CAF responders (*n* = 12), eight believed in the ergogenic effect of the supplement in question, and among the non-responders (*n* = 8), five reported having beliefs about the ergogenic effects of CAF. Regarding tDCS, of the ten responders, five believed in the effect of the stimulus in question, and the same occurred with the non-responders, in which 50% believed in the referred resource (*n* = 5). Among the ten participants who responded to the two treatments applied separately (CAF or tDCS), five of them reported believing in both treatments. Therefore, this suggests that 50% of the findings may have a potential placebo effect (CAF or tDCS).

### 3.6. Secondary Outcome

There was no significant relationship between the PR values relative to BM and the best performance in the RX Elite (r = 0.265; *p* = 0.526) and RX Intermediate (r = 0.049; *p* = 0.901) groups, suggesting that strength allometry is not a determining factor in C&J performance.

The duration of experience in the modality was associated with performance across different interventions. However, no significant associations were observed between the duration of experience and the interventions CAF + a-tDCS (r = −0.383; *p* = 0.129), PLA + a-tDCS (r = −0.442; *p* = 0.075), CAF + Sham-tDCS (r = −0.351; *p* = 0.167), and PLA + Sham-tDCS (r = −0.370; *p* = 0.144).

**Figure 8:**
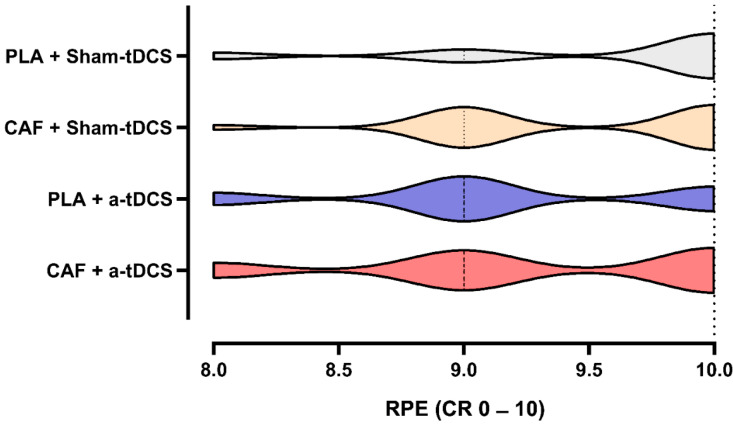
The median data of the RPE scale across interventions.

Finally, the RPE was compared across intervention types. After verifying the assumptions, the Friedman test was conducted to compare the median RPE scores among the different intervention types. As expected for a maximal time-trial protocol, no significant differences were observed between the interventions (CAF + a-tDCS = 9.0 [CI^95%^ 8.9–9.6]; PLA + a-tDCS = 9.0 [CI^95%^ 8.8–9.4]; CAF + Sham-tDCS = 9.0 [CI^95%^ 9.1–9.7]; PLA + Sham-tDCS = 10.0 [CI^95%^ 9.0–9.9]; *p* = 0.145). Figure 8 presents the median data of the RPE scale across interventions.

### 3.7. Relationship Between the Administered Caffeine (CAF) Dose and Performance

To ensure that the standardized CAF dose of 400 mg (5.2 ± 0.6 mg/kg) did not disproportionately benefit the participants with lower BM (i.e., those who received > 6.0 mg/kg) and consequently influence our results, a dose-response relationship analysis was conducted to exclude potential biases. Thus, the distribution of BM-relative CAF doses was assessed using the Shapiro-Wilk test, which confirmed normality (*p* = 0.351). Pearson’s correlation analysis did not reveal a significant association between the CAF dose and performance in the CAF + a-tDCS (r = 0.069; *p* = 0.794) or the CAF + Sham-tDCS (r = −0.053; *p* = 0.839).

### 3.8. Perceptions and Adverse Effects

Based on reports made by participants, there were no side effects resulting from the influence of tDCS. The perceptions arising from the tDCS intervention were recorded and are presented in Table 2. Regarding the CAF intake, there were no reports of muscle pain, increased urine output, tachycardia and heart palpitations, anxiety or nervousness, headache, gastrointestinal problems, and insomnia. However, it is important to note that two participants reported transient cardiac changes, which did not prevent them from performing the exercise.

It is worth noting that, in an attempt to minimize possible nocebo effects, we made a point of presenting the side effects and potential risks in a positive way, focusing on the benefits and highlighting the percentage of participants who did not experience adverse effects [28,29]. In cases of participants with some level of apparent or self-reported anxiety, Chamsi-Pasha et al. [28] recommends providing less information about mild or temporary adverse effects, in order to not potentiate the symptoms. We emphasize that the use of this strategy was clearly described in the informed consent form. On the other hand, we emphasize that it was not necessary to apply it to any of the participants. In addition, we were careful to use clear and understandable communication, suggesting examples to help them understand possible events and, in turn, manage their expectations [29]. Finally, we occasionally use counterconditioning techniques, which involve associating a positive or neutral stimulus with a negative or previously feared stimulus, which can favor the extinction of possible nocebo effects [28,29].

## 4. Discussion

The present study aimed to analyze the combined effects of tDCS and CAF on C&J performance in the CrossFit™ GRACE benchmark among competitive athletes. This study is pioneering, as it is one of the few in the literature to propose the ergogenic application of tDCS combined with CAF as a strategy to enhance sports performance in competitive CrossFit™ athletes. Our initial hypothesis suggested that tDCS and CAF alone would produce significant ergogenic effects on performance, and that the combination of tDCS and CAF would result in an additive effect, further improving execution time. However, the results of this study only partially supported this hypothesis, since tDCS and CAF alone did not produce effects on performance.

It is important to initially highlight some characteristics that may be defining for understanding our findings. It is well established that CrossFit™, also known as High-Intensity Functional Training (HIFT), has become a globally widespread sport, characterized by its multimodal approach that integrates various high-intensity exercises, Olympic weightlifting, and functional gymnastics [30,31]. The exponential growth of this practice has been driven not only by recreational engagement, but also by the rise in international competitions, such as the CrossFit™ games [30].

Athletes and coaches seek interventions and resources that enhance performance, aiming for competitive advantages. However, optimizing athletic performance in CrossFit™, across its various benchmarks, requires intensive investigation into different ergogenic aids, as the metabolic demands vary significantly between workout models [13,14]. For example, Meier et al. [13] compared the performance outcomes of the benchmarks GRACE, HELEN, and FRAN and observed that the physiological demands vary significantly among male athletes, as well as their completion times (233.3 ± 101.2 s; 611.2 ± 127.1 s; 310.4 ± 134.3 s, respectively, for GRACE, HELEN, and FRAN). Additionally, the study suggests that athletes with higher absolute strength have a greater likelihood of success in workouts predominantly based on weightlifting, such as GRACE. Therefore, additive effects on performance will depend on alignment with the type of ergogenic resource.

### 4.1. Primary Outcome

Our main finding highlights the combination of ergogenic strategies with distinct mechanisms of action. To date, no evidence adequately explains the effects of the combined use of CAF and tDCS, despite the well-established knowledge of their individual mechanisms. In this context, the leading hypothesis for the superiority of the combined intervention (CAF + a-tDCS) lies in the synergy between the neurophysiological mechanisms of these approaches. CAF, by blocking adenosine receptors, may enhance cortical activation induced by tDCS, facilitating synaptic transmission and prolonging neuronal excitability effects [2]. This synergistic effect may be associated with increased intracellular calcium availability and improved muscle contraction efficiency, as suggested by Kalmar and Cafarelli [32], who emphasized the CAF role in enhancing supraspinal muscle activation and mitigating central fatigue [33].

Furthermore, the reduction in perceived exertion induced by tDCS may be amplified by CAF central fatigue attenuation and enhancement of cognitive and attentional functions during exercise [9], although such an outcome was not observed in our study. Since the task involved a time-trial format, it is assumed that the effort level would naturally tend to its maximum throughout the performance.

One of the few studies available in the literature was conducted by Lattari et al. [4], that investigated the effects of the combination of tDCS and CAF on muscle strength and RPE during bench press exercises. The results indicated that all active conditions (tDCS and/or CAF) increased muscle strength compared to the placebo (Sham + PLA: 661.2 ± 112.6 kg; Sham + CAF: 806.9 ± 160.4 kg; a-tDCS + PLA: 800.7 ± 139.3 kg; a-tDCS + CAF: 842.1 ± 177.3 kg; *p* < 0.001). However, only the combined condition (a-tDCS + CAF) significantly reduced RPE compared to PLA (*p* < 0.05). These findings suggest that the combination of tDCS and CAF may synergistically modulate RPE, enhancing the ergogenic effects of both strategies through cortical excitation and adenosine blockade.

However, it is clear to us that the task proposed by Lattari et al. [4] is of low complexity, performed under stable conditions, and does not require cognitive regulation for its successful execution. In contrast, in our study, the C&J movement represents a complex sequence of motor actions, including deadlift, front squat, push jerk or power jerk, executed in a continuous and uninterrupted manner until exhaustion [13,16]. This demands intrinsic prediction based on cognitive processes, requiring athletes to develop a strategic regulation of effort throughout the open-ended time trial [34]. Therefore, despite the similarity in intervention design, the results of Lattari et al. [4] cannot be extrapolated to other conditions, such as those observed in the WOD GRACE.

In isolation, the significant improvement in performance following CAF supplementation is well-documented in the literature [2,14,17,18]. Studies by Grgic [3] and Bilondi et al. [17] indicate that CAF enhances neuromuscular efficiency, reduces perceived exertion, and increases power output in resistance-based and intermittent high-intensity exercises. However, in the context of CrossFit™, CAF supplementation has shown mixed results, with studies like Fogaça et al. [35] failing to observe an impact. Our study is in line with Fogaça et al. [35], demonstrating no significant effect on WOD GRACE (alone). Despite this, there are substantial differences between the workouts used. Similarly to our study, Ziyaiyan et al. [15] evaluated the effects of CAF, sodium bicarbonate (NaHCO_3_), and their combination (CAF + NaHCO_3_) on performance and physiological factors in CrossFit™ practitioners during the Cindy WOD. The ergogenic aids did not result in a significant improvement in the total number of repetitions completed with CAF (71.59 ± 439.60), NaHCO_3_ (65.56 ± 432.95), or CAF + NaHCO_3_ (75.96 ± 445.35), compared to the placebo (70.42 ± 428.20) and control (68.34 ± 419.50). However, the combined supplementation of CAF + NaHCO_3_ significantly reduced the RPE (CAF—6.06 ± 0.58, compared to NaHCO_3_—6.41 ± 0.48, and CON—6.75 ± 0.48), thus differing from our results.

In this case, CAF is widely recognized for its ergogenic effects, particularly in reducing the perception of effort across various sports modalities. This effect is primarily mediated by its role as an antagonist of adenosine A1 and A2A receptors in the central nervous system, which also contributes to increased alertness and enhanced neuromuscular function [2,3,17]. Furthermore, evidence suggests that CAF influences corticomotor excitation by enhancing corticospinal tract excitability and facilitating the recruitment of motor units [14,19].

Cristina-Souza et al. [33] provided novel evidence regarding the neural and muscular mechanisms by which CAF enhances performance during high-intensity exercise. The study demonstrated that the ingestion of 5 mg/kg of CAF (same dose used in our study) increased time to exhaustion by 14.4% (*p* = 0.017) during whole-body exercises, without altering the rate of muscular fatigue. These effects were attributed to the CAF ability to attenuate the decline in muscular contractile strength, as indicated by the preservation of twitch force in the quadriceps (*p* = 0.014). Furthermore, supplementation maintained peripheral oxygenation by keeping oxygen saturation at significantly higher levels (95.0 ± 1.9%) compared to placebo (92.0 ± 6.2%, *p* = 0.016), suggesting an improvement in oxygen delivery to active muscles. Additionally, CAF reduced the decline in voluntary activation throughout exercise (–0.5 ± 4.2% vs. –5.8 ± 8.5% in the placebo group, *p* = 0.004), indicating preserved motor excitability. This effect may be particularly relevant for activities requiring high force production and endurance, such as CrossFit™ WODs, where the ability to sustain repeated, intense efforts is a critical determinant of performance.

In parallel, we found no significant differences in the intervention of tDCS alone. This finding refutes our primary hypothesis that neuromodulation would directly improve performance through cortex excitability enhancement. It is worth highlighting that as this is a pioneering study, comparisons become difficult, requiring inference based on similar modalities. We know that the lack of significant improvements in performance with tDCS alone aligns with previous studies that questioned its efficacy in short-duration, high-intensity tasks. Studies by Teymoori et al. [36] and Angius et al. [37] found that tDCS failed to enhance anaerobic performance but showed positive effects on endurance-based activities.

Several factors may explain the lack of tDCS-induced improvements. The workout GRACE benchmark is a short-duration, maximal-effort and closed-task. Studies suggest that tDCS may be more effective for longer-duration endurance tasks rather than explosive, strength-based exercises [7]. Yu et al. [7] conducted a systematic review to evaluate the effects of tDCS on national- and international-level athletes, exploring its impact on both the physical and psychological aspects of sports performance. The authors analyzed 20 experimental studies involving 224 participants, identifying significant improvements in strength (67% of studies), endurance (75%), and emotional states (75%), whereas the effects on sport-specific tasks (40%) and cognitive performance (33%) were more inconsistent. According to the authors, “tDCS is an effective tool that can be applied in competitions and pre-competitive training to enhance athletic performance”.

On the other hand, Lattari et al. [38] examined the impact of a-tDCS, cathodal tDCS (c-tDCS), and PLA on strength-trained individuals, demonstrating that a-tDCS applied to the DLPFC significantly increased the volume load in the leg press exercise compared to the other conditions (F(2,28) = 164.801; *p* < 0.001). Additionally, the perceived exertion (OMNI-RES) score was higher in the c-tDCS condition compared to a-tDCS and placebo (F(2,28) = 9.768; *p* < 0.05), suggesting that a-tDCS stimulation may mitigate central fatigue and enhance muscular endurance during strength training. Once again, we emphasize that task complexity appears to be a key factor in distinguishing our results.

Regarding the analysis of responders and non-responders to active treatments (tDCS and CAF), when analyzing tDCS in isolation, 50% (*n* = 10) of the sample responded positively to the intervention, showing a reduction in total performance time. Whereas, for the CAF treatment, 60% (*n* = 12) of the participants exhibited a positive response after said treatment. For a more in-depth analysis, responders were stratified based on the 50th percentile into superior performance (shortest performance times, i.e., less than 240 s) and inferior performance (longest performance times, i.e., greater than 240 s), both for the tDCS intervention alone and for CAF supplementation. Among the ten participants responding to the tDCS intervention, only three were classified as high performers (shortest performance times), while seven were classified as low performers (longest performance times). Participants in the highest performance stratum appeared to benefit most from the tDCS intervention, showing an average performance improvement of approximately ∆% = −20% after stimulation, while the lowest performance stratum only exhibited a change of approximately ∆% = −10%. For the CAF treatment, twelve participants responded positively to supplementation. Among them, five participants were classified as the highest performance stratum (performance times less than 231 s) and seven were categorized as lowest performance (performance times greater than 231 s). In this case, the opposite pattern was observed in the tDCS treatment. Participants in the lowest performance stratum responded more strongly to CAF supplementation (∆% = −11%), while those in the highest performance stratum showed a smaller change in performance (∆% = −7%). In summary, for the tDCS treatment, the modulation in performance appears to be more evident in subjects with higher performance. This can be partly explained, perhaps because these (more trained) subjects are more sensitive to neural stimuli. Kleemeyer et al. [39] demonstrated that greater changes in physical fitness (VO_2_max and VO_2_ at the anaerobic threshold) are associated with a greater change in neural plasticity (r = 0.310, *p* = 0.036). It is reasonable to infer that greater neural plasticity may also be associated with greater neural sensitivity, as there is more nerve area to receive the stimulus. On the other hand, Warren et al. [40], in a systematic review and meta-analysis study, although they demonstrated that the CAF treatment has an effect on trained and untrained subjects, trained subjects showed a smaller magnitude of the treatment effect (ES = 0.13) compared to untrained subjects (ES = 0.21).

Regarding the results obtained based on the interview to transmit the results to each of the participants through an informal interview, but based on a semi-structured questionnaire (Supplementary Materials), the results indicated that among the CAF responders (*n* = 12), eight believed in the ergogenic effect of the supplement in question, and among the non-responders (*n* = 8), five reported having beliefs about the ergogenic effects of CAF. Regarding tDCS, of the ten responders, five believed in the effect of the stimulus in question, and the same occurred with the non-responders, in which 50% believed in the referred resource (*n* = 5). Among the ten participants who responded to the two treatments applied separately (CAF or tDCS), five of them reported believing in both treatments. Thus, suggesting that 50% of the findings may have a potential placebo effect (CAF or tDCS). Regarding the use of CAF alone, eight of the twelve CAF respondents reported believing in the effects of the supplement in question, thus conferring a potential placebo effect of 66.7%. Beedie et al. [41], who investigated only the use of CAF, detected a placebo effect in seven of the five individuals, thus reporting a placebo effect of 71.4%, reasonably similar to that presented in our study. Regarding this type of analysis with the use of tDCS, to the best of our knowledge, it has not yet been carried out, which prevents us from discussing this point. However, it is worth noting that this analysis goes beyond the scope limit that the present experiment intended.

### 4.2. Secondary Outcome

We emphasize that our secondary hypothesis was also refuted, as RPE did not decrease under the influence of either the combined intervention strategies or the isolated conditions. Previous studies have demonstrated that both CAF and tDCS can reduce RPE, facilitating exercise performance at higher intensities and for extended durations [2,3,38,42]. However, these effects do not appear to manifest similarly in complex, short-duration and closed-tasks (time trial), such as the neuromuscular demands of the C&J movement and because it is a closed-ended task protocol, i.e., time trials. The DLPFC was selected as the tDCS stimulation target based on its role in cognitive processes related to effort modulation, with the aim of inhibiting mechanisms that might contribute to effort perception [5,6,42,43]. Although, with regard to tDCS alone, a recent and elegant systematic review of Baharlouei et al. [9] demonstrated that, of the nine studies that used the same scale of perceived exertion as the present study, seven demonstrated no reduction [43,44,45,46,47,48,49], one demonstrated an increase [50] and only one demonstrated a reduction in the RPE [51]. Thus, it may be that the reduction in the RPE associated with tDCS is scale dependent. Regarding CAF supplementation alone and its effects on the same variable (RPE), a recent systematic review with meta-analysis [52], demonstrated that CAF does not appear to alter RPE. We emphasize that the effects of tDCS or CAF supplementation on the RPE may also be affected by the nature of the exercise investigated. Apparently, open-task resistance exercises, as well as continuous and aerobic exercises may be more sensitive in detecting changes in the RPE when using tDCS and/or CAF [9,52]. On the other hand, we had hypothesized that the combination of the two ergogenic resources we tested (tDCS and CAF) could be efficient in producing a significant reduction in the RPE. Finally, although the study by Lattari et al. [4] found a significant reduction in the RPE when associating tDCS with CAF, to our knowledge, there is no other study in the literature that has carried out a similar experiment and, possibly, the difference with the present study may have been due to the different characteristics of the protocols, as previously mentioned (complexity and neuromuscular demands of the C&J movement and closed-ended task protocol). However, if we analyze the evidence of isolated use of the different ergogenic resources tested (tDCS or CAF), the results of the present study on the variable in question seem to be in line with the body of literature presented so far.

Regarding performance, notably, the combination of a-tDCS and CAF resulted in significant performance improvements, reinforcing the idea that the CAF antagonistic effect on adenosine receptors may potentiate the excitatory impact of tDCS on motor cortex function. Additionally, our findings suggest that in high-intensity, resistance-endurance, and short-duration exercises, isolated tDCS may not be sufficient to elicit ergogenic effects. However, when combined with CAF neuromodulatory properties, it may induce a synergistic impact on neuromuscular efficiency, though not on effort regulation in short-duration trials. This discrepancy highlights the need for future research to explore the role of tDCS in multimodal high-intensity protocols, where cognitive load, motor complexity, and strategic pacing may influence its effectiveness.

Finally, PR values adjusted for BM did not yield the expected results, considering the perspective presented in the literature. Albernaz et al. [16] analyzed whether PR, anthropometric variables, and experience could effectively predict strength-endurance performance in the C&J movement, specifically within the GRACE benchmark of CrossFit™. The results demonstrated a significant association between PR and GRACE performance time (r = −0.690; R^2^ = 0.482; *p* = 0.001), indicating that PR explained 48% of the performance variance. However, anthropometric variables such as lean mass (r = −0.314; *p* = 0.220), fat percentage (r = 0.274; *p* = 0.228), and training experience (r = −0.414; *p* = 0.098) showed no significant correlation with performance in GRACE. These findings suggest that PR is a moderate predictor of strength endurance in C&J, while morphological factors and training duration do not appear to play a determining role. Other evidence supports the same perspective. For example, total body strength, measured individually through PR in the Press, Squat, and Deadlift, adequately predicts C&J performance (R^2^ = 0.77; *p* = 0.0001) [53].

The findings of Meier, Schlie and Schmidt [54] revealed that physiological factors, such as body composition, aerobic capacity (VO_2_max), and muscular strength, appear to be key contributors to performance. Specifically, a lower body fat percentage correlated with better performance in the CrossFit™ open and endurance-based WODs (Murph, Donkey Kong) (r = 0.72; *p* < 0.05), while VO_2_max exhibited moderate to high correlations with performance in aerobic-demanding WODs (r = 0.68 to 0.81; *p* < 0.05). The GRACE WOD stands out from other CrossFit^®^ protocols, demonstrating that absolute strength in the squat and C&J significantly correlates with WODs incorporating weightlifting elements (FRAN, GRACE) (R^2^ = 0.42 to 0.84; *p* < 0.05). However, to our knowledge, no study has examined this relationship when adjusted for BM, making direct comparisons challenging.

Experience in high-level competitions was identified as another strong predictor of CrossFit™ performance in Meier, Schlie and Schmidt [54]. In our study, competitors exhibited heterogeneity in prior competition experience, as they participated primarily in regional and state-level competitions, with limited involvement in national-level events (Brazil). While predictions based on CrossFit™ open rankings using experience as a variable explained 39% of the variance in competition results in the study by Meier et al., which was not observed in our study.

### 4.3. Limitations

Several limitations should be considered: (a) Interindividual variability in response to tDCS may influence the effectiveness of stimulation, as factors such as cortical thickness, training level, and genetic predisposition can impact neuronal excitability and the ergogenic response to the technique [7]. (b) The lack of significant effects from isolated tDCS may be related to the need for prolonged stimulation protocols, similar to those observed in endurance tasks, which tend to produce more robust effects [10]. (c) The CAF dose administered could introduce a prescription-related bias; however, the lack of a dose-response association strengthens our findings. (d) Although the sample size was calculated to ensure adequate statistical power, it may still have contributed to the retention of the null hypothesis for individual outcomes.

## 5. Conclusions

The combination of CAF with tDCS significantly improved performance in the GRACE benchmark, reducing the total execution time of the C&J. However, the isolated interventions of tDCS or CAF did not show significant differences compared to the placebo. The responder analysis indicated that 50% of participants experienced performance improvements with tDCS, whereas 60% responded positively to CAF. No significant differences were observed in the RPE across interventions.

Regarding the informal interviews conducted to share individual results with participants, 66.7% of CAF responders believed in the supplement’s ergogenic effects, while 62.5% of non-responders also held this belief. Similarly, 50% of both tDCS responders and non-responders believed in the efficacy of the stimulation. Among the ten participants who responded positively to either intervention (CAF or tDCS), half believed in both. These findings suggest that up to 50% of the observed effects may be influenced by placebo responses. While this interview-based analysis was not an outcome variable of the study, it was conducted to provide participants with personalized feedback and to foster informed decisions about ergogenic strategies. We recommend interpreting these results with caution, as they extend beyond the original scope of the study. Nonetheless, we consider it valuable to report them to encourage further research on placebo effects in performance enhancement.

In the secondary analyses, no significant correlation was found between PR values adjusted for BM and WOD GRACE execution time in either the RX Elite group or the RX Intermediate group. Additionally, experience in the modality did not show a significant association with performance across the different experimental protocols.

These results indicate that tDCS, when combined with CAF, may serve as an effective ergogenic aid for enhancing CrossFit™ performance in purely strength workouts, whereas its isolated use does not provide relevant benefits for this type of workout.

## Figures and Tables

**Table 1:** Sample characterization (*n* = 20).

	Age	Exp	Body Mass	Height	BMI	Fat	Lean Mass	Total Water
	(Year)	(Months)	(kg)	(cm)	kg/m^2^	(%)	(kg)	(L)
Mean	31.1	49.0	77.3	1.73	26.0	17.0	36.1	46.2
SD	5.0	30.0	12.3	0.08	2.9	8.3	5.7	7.0
CI^95%^	28.5–33.7	33.6–64.4	70.9–83.6	1.68–1.77	24.4–27.4	13.5–22.1	33.1–39.0	42.6–49.8

Exp = time of experience; BMI = body mass index; % fat = percentage fat; BMI and Fat% were treated non-parametrically, therefore, the reported values are median.

**Table 2:** Adverse effects of tDCS—sensory perception.

Parameters	Percentage (%)
Itching	70.0%
Pain	5.5%
Burning Sensation	50.0%
Warmth	5.56%
Tingling	100.0%
Fatigue	0.0%
Other	0.0%

## Data Availability

Data are contained within the article and will be made available upon request.

## Supplementary Materials

The following supporting information can be downloaded at: https://www.mdpi.com/article/10.3390/nu17071261/s1, Table S1: Pre Intervention-Suggested Interview Procedure; Table S2: Post Intervention.

## Abbreviations

The following abbreviations are used in this manuscript:

CAF	Caffeine
tDCS	Transcranial Direct Current Stimulation
PLA	Placebo
PR	Personal Record
RPE	Rating of Perceived Exertion
RX	Prescribed Exercise Standard in CrossFit™
DLPFC	Dorsolateral Prefrontal Cortex
VO_2_max	Maximal Oxygen Consumption
M1	Primary Motor Cortex
BIA	Bioelectrical Impedance Analysis
BM	Body Mass
BMI	Body Mass Index
CAD	Coronary Artery Disease
NaHCO_3_	Sodium Bicarbonate
ES	Effect Size
CI95%	95% Confidence Interval
ACSM	American College of Sports Medicine
SPSS	Statistical Package for the Social Sciences
G*Power	Statistical Power Analysis Software
ICMJE	International Committee of Medical Journal Editors
STROBE	Strengthening the Reporting of Observational Studies in Epidemiology
